# Morphometric and Clinical Analysis of the Azygos Anterior Cerebral Artery: Insights From a Cadaveric Case Report Study

**DOI:** 10.7759/cureus.83063

**Published:** 2025-04-27

**Authors:** Gervith Reyes Soto, Carlos Castillo-Rangel, Pablo Gonzalez-Lopez, Tshiunza Mpoyi Cherubin, Vladimir Nikolenko, Andreina Rosario Rosario, Amaya Alvarez Aquino, Iselle Suero Matos, Nasser M F El-Ghandour, Manuel De Jesus Encarnacion Ramirez

**Affiliations:** 1 Neurosurgical Oncology, Mexico National Cancer Institute, Tlalpan, MEX; 2 Neurosurgery, Service of the 1ro de Octubre Hospital of the ISSSTE, Mexico City, MEX; 3 Neurosurgery, Hospital General Universitario de Alicante, Miguel Hernandez University, Alicante, ESP; 4 Neurosurgery, Clinique Ngaliema, Kinshasa, COD; 5 Human Anatomy and Histology, N.V. Sklifosovskiy Institute of Clinical Medicine, Moscow, RUS; 6 Medicine, Autonomous University of Santo Domingo (UASD), Santo Domingo, DOM; 7 Neurotrauma and Global Neurosurgery, Foundation Meditech, Cali, COL; 8 Medicine, Instituto Tecnologico de Santo Domingo, Santo Domingo, DOM; 9 Neurosurgery, Faculty of Medicine, Cairo University, Cairo, EGY; 10 Neurosurgery, Russian People’s Friendship University, Moscow, RUS

**Keywords:** anterior cerebral artery, anterior communicating segment, azygos, fronto-basal trunk, neuroanatomy

## Abstract

The azygos anterior cerebral artery (ACA) is an uncommon anatomical variation with important clinical implications. This study presents an in-depth morphometric analysis of a single brain specimen displaying this variant. The goal is to improve the understanding of its anatomical characteristics and its influence on cerebral vascular health. Through precise dissection and measurement, the study highlights the structural features of the azygos ACA and its potential role in clinical conditions, particularly in relation to aneurysm formation and associated risks. The brain specimen was sourced from the Laboratory of Microsurgical Anatomy of the Central Nervous System, Faculty of Medicine, National Autonomous University of Mexico (UNAM). The donor did not have a history of neurological disease or neurosurgical procedures, ensuring the integrity of the vascular structures for analysis. To facilitate analysis, red latex was injected into the specimen, which was then preserved in 10% formalin for two months. Morphometric assessments were carried out using a Mitutoyo Model CD-8” CX digital caliper with a resolution of 0.0005”/0.01mm, along with detailed photographic documentation. The study examined the brain of a 38-year-old male, which weighed 1,310 grams. Symmetry was observed in the A1 segments: the right A1 segment measured 11 mm in length and 3 mm in diameter, while the left A1 segment measured 10 mm in length and 2.4 mm in diameter. The anterior communicating artery (ACoA) was absent, and only a single A2 trunk was identified. This trunk had an initial diameter of 4 mm, which tapered to 3 mm over a length of 33 mm until it bifurcated. Common anatomical variations of the A1-ACoA complex include absence, bifurcation, hypoplasia, plexal arrangement, and fenestration. A thorough understanding of anterior cerebral circulation variants is essential for optimizing the treatment of vascular pathologies.

## Introduction

The azygos anterior cerebral artery (ACA) is a rare anatomical variant with an incidence of 1.1%. This artery is also referred to by several names, including terminal artery, common trunk of the cerebral artery, azygos pericallosal artery (PCA), unpaired PCA, and unpaired cerebral artery [1,2]. The ACA originates from the internal carotid artery, anterior to the olfactory trigone and optic chiasm [1]. Following a short horizontal and anteromedial course, it travels along the base of the optic chiasm and enters the interhemispheric fissure. Before reaching the fissure, it connects with its counterpart through the anterior communicating artery (ACoA) (A1 segment). The distal portion of the ACA, also known as the PCA, is divided into four segments: A2 segment, which extends from the ACoA to the rostrum of the corpus callosum (CC); A3 segment, which runs from the rostrum of the CC to the point where it turns horizontally at the genu of the CC; A4 segment, which extends from the genu to a line perpendicular to the foramen of Monroe or the coronal suture; and A5 segment, which continues from the previous point to the splenium of the CC [2,3].

The clinical significance of the azygos ACA stems from its association with neurological conditions, particularly an increased risk of aneurysms in regions where the artery divides [4]. Aneurysms in this variant pose unique surgical challenges due to the complex vascular anatomy and the potential presence of congenital malformations, such as agenesis of the CC or arteriovenous malformations (AVMs) [5,6].

The embryological development of the azygos ACA offers valuable insights into its formation and potential anomalies. During early embryogenesis, cerebral vascular patterns undergo dynamic changes. Initially, the ACA forms as a single vessel in early embryogenesis, which then divides into two to supply the cerebral hemispheres [6]. Failure of this bifurcation process results in the persistence of a single-trunk ACA, termed “azygos ACA” [7,8]. Genetic and environmental factors influencing vascular signaling pathways are thought to contribute to this anomaly, though the precise etiology remains unclear [8,9].

Hemodynamic alterations caused by the azygos ACA have significant clinical implications. Abnormal blood flow patterns and increased vascular wall stress predispose this variant to aneurysm formation, often at atypical locations [10-12]. Ruptured aneurysms can result in subarachnoid hemorrhage, a life-threatening condition, emphasizing the importance of detailed anatomical knowledge for neurosurgeons and radiologists [10,11].

Advanced imaging modalities, such as magnetic resonance angiography (MRA), computed tomography angiography (CTA), and digital subtraction angiography (DSA), are essential for identifying and evaluating the azygos ACA and related aneurysms [10,11]. Accurate imaging helps clinicians avoid misdiagnosis and plan safer interventions.

The presence of an azygos ACA also complicates surgical and endovascular procedures. Awareness of this variant is crucial during aneurysm clipping, coiling, or unrelated surgeries to prevent inadvertent vessel damage. Moreover, the azygos ACA is sometimes associated with additional vascular anomalies, such as AVMs, further complicating treatment strategies [11,12].

The altered hemodynamics linked to the azygos ACA can affect cerebral perfusion, potentially causing areas of hypoperfusion or hyperperfusion. Understanding these dynamics is critical for managing cerebrovascular diseases and predicting therapeutic outcomes [9-11].

This study highlights the morphometric characteristics and clinical implications of the azygos ACA, emphasizing its importance in diagnostic and therapeutic contexts. Recognizing this variant in clinical practice is vital for optimizing patient care and reducing complications.

## Case presentation

Specimen preparation

The brain of a 38-year-old male, which weighed 1,310 grams, was analyzed at the Laboratory of Microsurgical Anatomy of the Central Nervous System, Faculty of Medicine, National Autonomous University of Mexico (UNAM). The donor's cause of death was non-neurological. The donor did not have a history of neurological disease or neurosurgical procedures, ensuring the integrity of the vascular structures for analysis.

Initial treatment

The cerebral arterial system was perfused with 0.9% saline solution through a latex catheter for 24 hours immediately after extraction. Continuous irrigation of both carotid and vertebral arterial systems was performed to remove residual blood and prevent clot formation.

Latex injection

To enhance vascular visualization, the right carotid and vertebral arteries were ligated using 3/0 silk sutures. Red latex was injected into the left carotid artery system and subsequently into the left vertebral arterial system, which was then ligated. This dual-injection method ensured thorough filling of the arterial system for detailed morphometric analysis [13].

Fixation

The brain was then fixed by immersion in 10% formalin for two months. This fixation process preserved the tissue structure and maintained the integrity of the vascular system, essential for subsequent detailed dissection and analysis.

Microsurgical dissection

A meticulous dissection of the anterior cerebral arterial system, including segments A1 through A4: the A1 segment was identified on both sides, coursing medially and slightly anteriorly from the internal carotid artery toward the ACoA, the A2 segment was identified as the single trunk extending from the confluence of the A1 segments, characteristic of the azygos ACA, the A3 segment begins at the genu of the CC and represents the continuation of the azygos ACA of the ACA, was carried out, and the A4 segment was carefully traced as it extended posteriorly along the CC. The procedure was performed under a Carl Zeiss OPMI™ surgical microscope with magnifications ranging from 6x to 40x. Microsurgical scissors and watchmaker's forceps no. 3 were employed for precision and to handle delicate vascular structures.

Morphometric analysis

Arterial measurements were taken using a Mitutoyo Model CD-8” CX digital caliper, offering a resolution of 0.0005”/0.01mm, ensuring high precision. Supplementary recordings and cross-verification of measurements were performed using graph paper, with each square on the graph paper measuring precisely 1 mm x 1 mm, providing a standardized grid for accurate visual scaling and manual measurement.

Documentation

Morphological and morphometric data were meticulously documented. High-resolution photographs were captured using an Olympus™ μ DIGITAL 800 camera (Olympus Corporation, Tokyo, Japan) equipped with an 8.0-megapixel sensor to preserve anatomical details for analysis and reporting.

**Figure 1:**
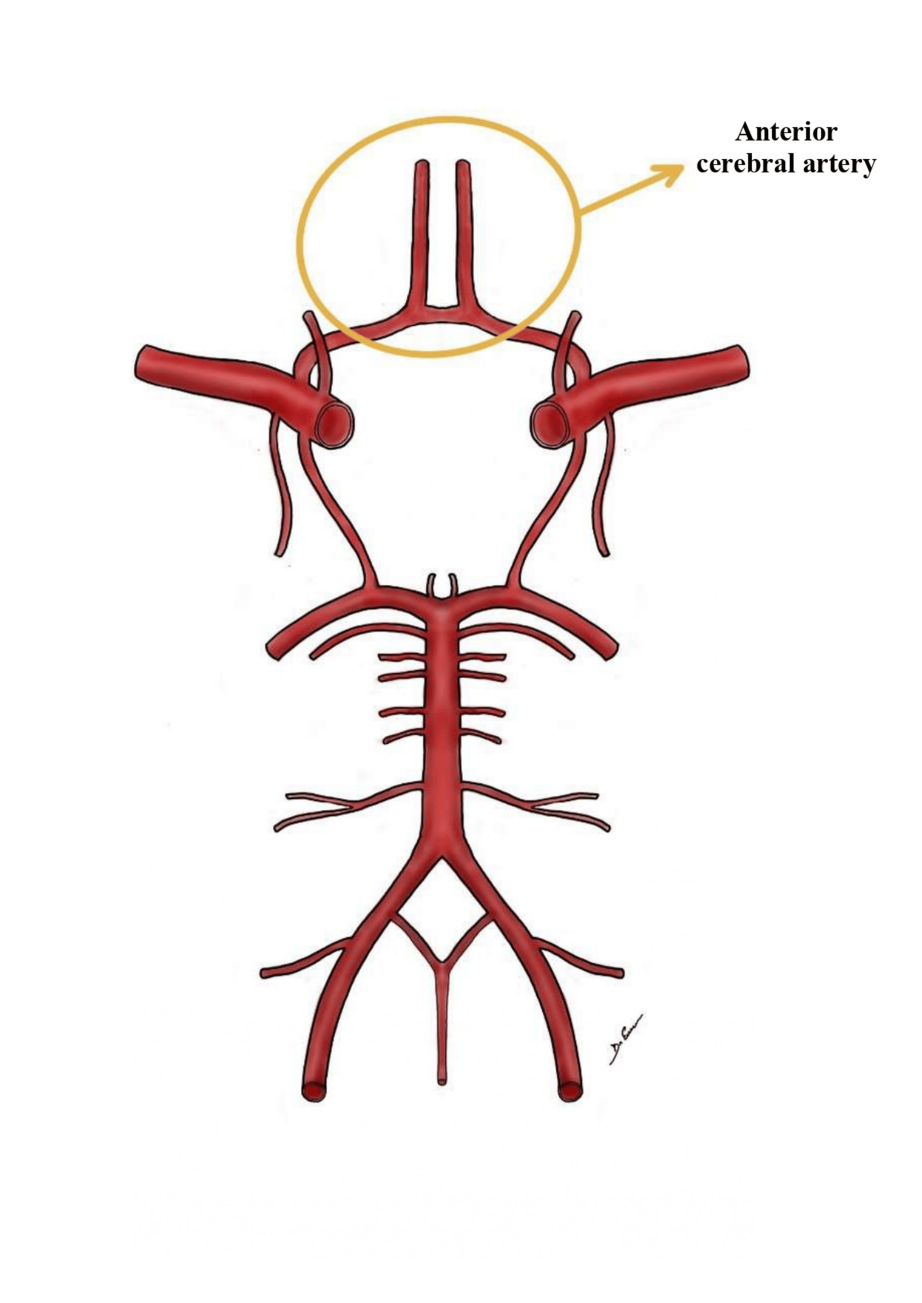
Original illustration depicting the anterior cerebral artery (ACA), the anterior communicating artery (ACoA), and the A2 segment. ACA, anterior cerebral artery

**Figure 2:**
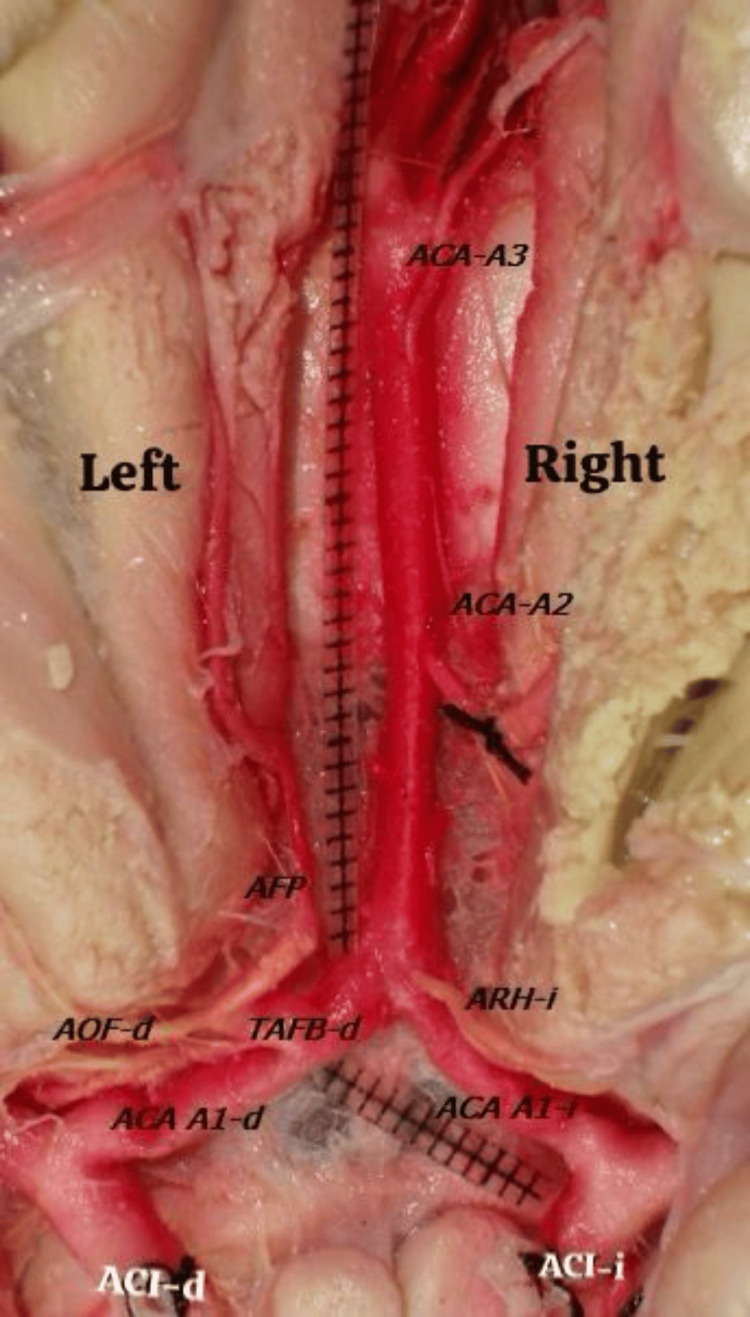
Microphotograph of azygos ACA. ACA, anterior cerebral artery; ACA-A3, anterior cerebral artery A3 segment; ACA-A2, ACA segment A2; AFP, frontopolar artery; TAFB-d, right frontobasal arterial trunk; AOF-d, right orbitofrontal artery; ARH-i, left recurrent artery of Heubner; ACA A1-d, right ACA segment A1; ACAA1-i, anterior cerebral artery A1 segment; ACI-d, right internal carotid artery; ACI-i, left internal carotid artery.

The A1 segments of the ACA originated bilaterally from the medial surface of the internal carotid artery (Figure 1), ventral to the olfactory trigone. Each segment traveled horizontally in an anteromedial direction, passing rostrally to the optic chiasm and entering the interhemispheric fissure to form a single A2 segment, which indicates an azygos ACA. The right A1 segment (A1-d) measured 11 mm in length and 3 mm in external diameter, while the left A1 segment (A1-I) measured 10 mm in length and 2.4 mm in diameter (Figure 2).

**Figure 3:**
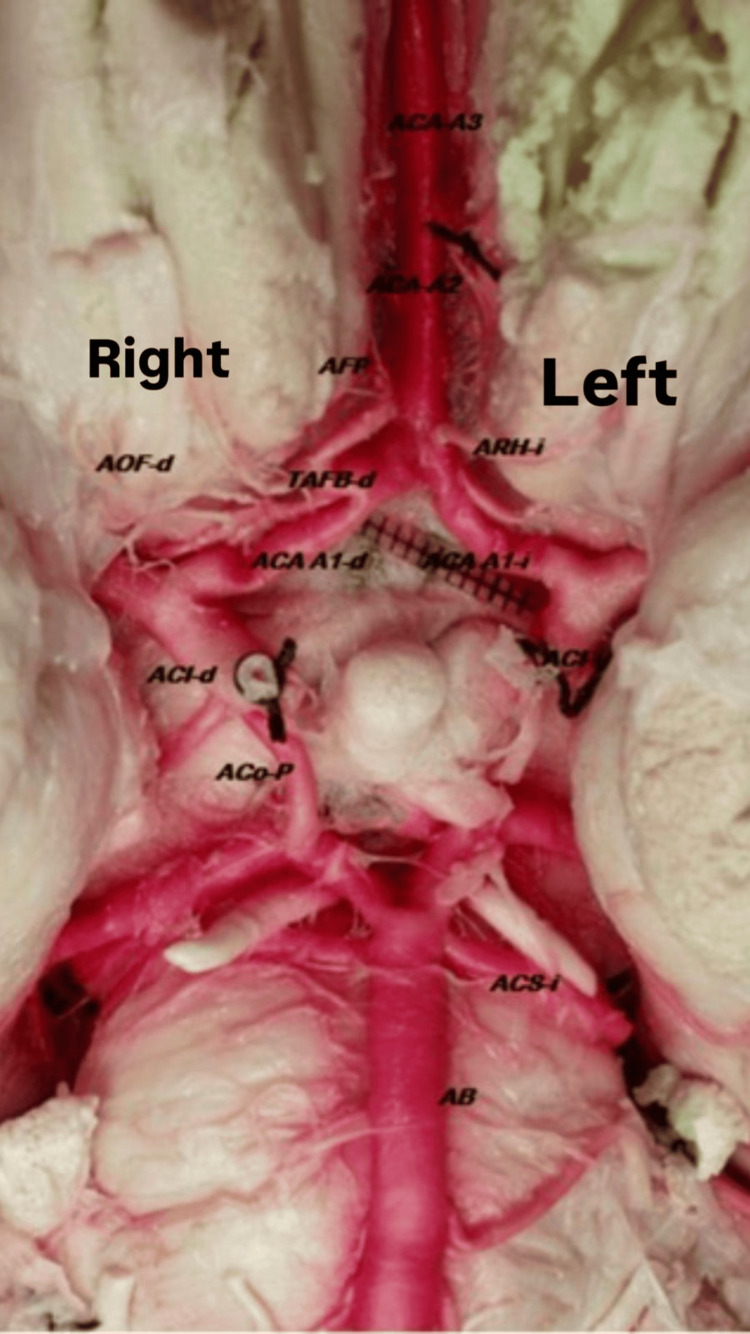
Azygos ACA microphotography. ACA, anterior cerebral artery; ACA-A3, anterior cerebral artery A3; ACA-A2, ACA segment A2; AFP, frontopolar artery; TAFB-d, right fronto-basal arterial trunk; AOF-d: right orbitofrontal artery; ARH-i, left recurrent Heubner's artery; ACA A1-d, right ACA segment A1; ACA A1-i, left ACA segment A1; ACI-d, right internal carotid artery; ACI-i, left internal carotid artery; Aco-P, posterior communicating artery; ACS-I, left superior cerebellar artery; AB, basilar artery

Perforating branches from the lateral borders of the A1 segments supplied the rostrum, lamina terminalis, anterior commissure, optic chiasm, and paraolfactory area. The A1-d segment had 11 perforating branches with diameters ranging from 0.005 mm to 1.90 mm, while A1-I had 14 branches with diameters ranging from 0.006 mm to 1.10 mm (Figure 3). The left recurrent artery of Heubner (ARH-i) originated from the lateral border of A1 and measured 1.20 mm in diameter and 14 mm in length, directed towards the anterior perforated substance (Figure 3). A distinct right frontobasal trunk (TFB-d) was observed 10 mm from the A1 segment of the right ACA, measuring 5 mm in length and 2 mm in diameter. This trunk gave rise to the following: a) right orbitofrontal artery (AOF-d), which was 13 mm long and 1 mm in diameter, with no perforating branches, b) right frontopolar artery (AFP-d)), which was 16 mm long and 2.1 mm in diameter, also without perforating branches, c) right recurrent artery of Heubner (ARH-i)), which is a single trunk measuring 1.3 mm in diameter, bifurcating 12 mm from its origin into two branches of 0.8 mm and 0.9 mm, directed towards the anterior perforated substance.

**Figure 4:**
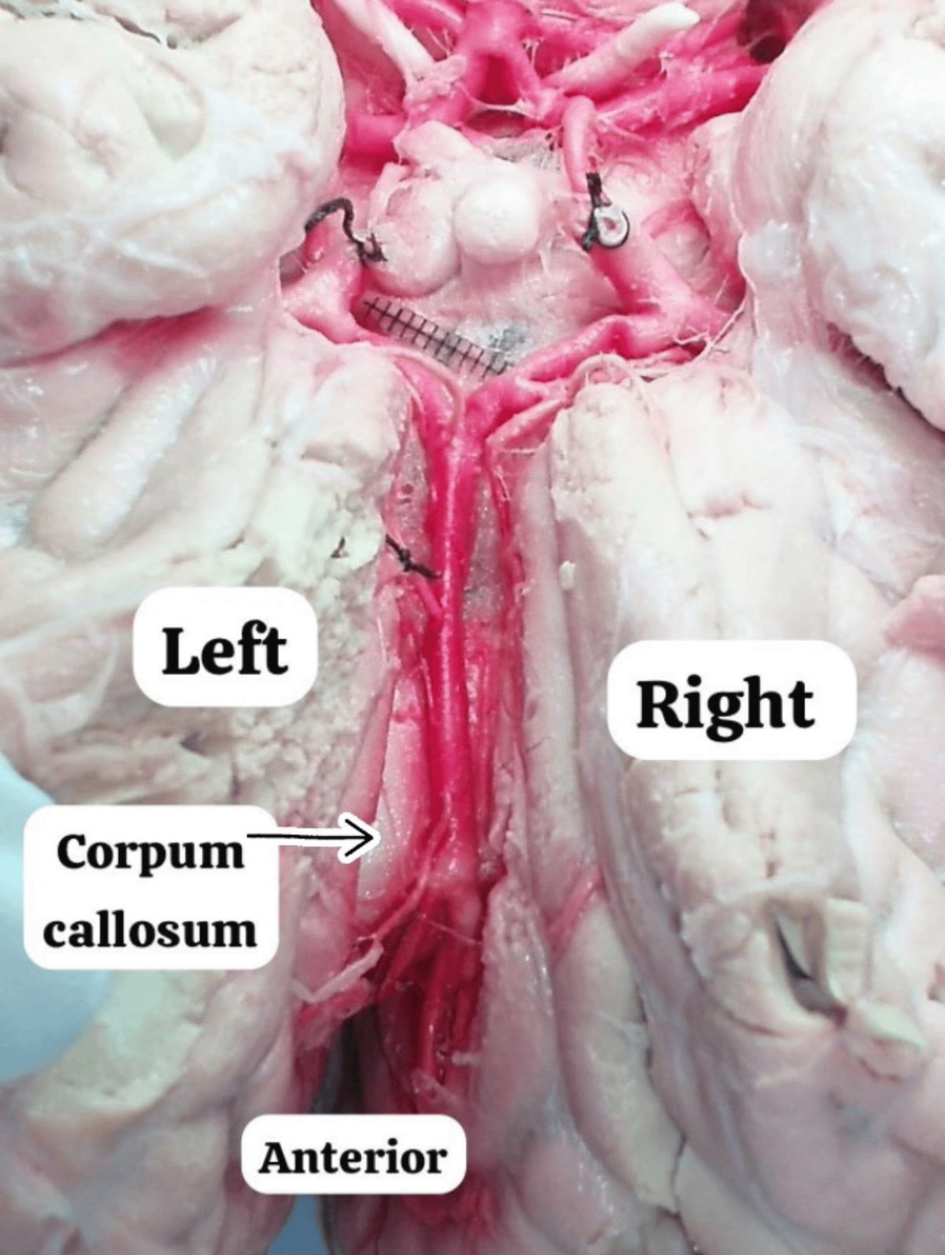
Cadaveric dissection illustrating the azygos anterior cerebral artery and its anatomical relationships within the interhemispheric sulcus.

The ACoA was absent. A single A2 trunk, initially 4 mm in diameter, tapered to 3 mm over a length of 33 mm before bifurcation. This trunk entered the interhemispheric fissure and progressed rostrally into the cistern of the corpus callosum (CC), continuing as the A3 segment, anterior to the genu of the CC (Figure 4).

 The bifurcation of the A3 segment of the azygos anterior cerebral artery (ACA) is located near the genu of the corpus callosum (CC). This variant corresponds to type II azygos ACA. The A3 segment bifurcated into right and left A3 segments 33 mm from the A2 origin. Both A3 segments measured 3 mm in diameter and gave rise to the pericallosal and callosomarginal arteries bilaterally. The right ACaM measured 3.3 mm in diameter and 37 mm in length, while the left ACaM measured 3.7 mm in diameter and 36 mm in length. The right pericallosal artery (A-PeCd) measured 2.2 mm in diameter and 44 mm in length, and the left pericallosal artery (A-PeCi) measured 2.6 mm in diameter and 45 mm in length (Table 1).

**Table 1:** ACA branches distance and diameter ACA, anterior cerebral artery. Note: The asterisk (*) in the perforating (number) column indicates that the number of perforators was not determined due to anatomical variability or limited visibility during dissection.

Branches	Diameter (mm), mean ± SD	Length (mm) mean ± SD	Perforating (number)	p-Value
A1-d	4 ± 0.5	14 ± 1.2	12 ± 1.0	0.01
A1-i	2 ± 0.3	16.72 ± 1.4	18 ± 1.3	0.02
AR-Hubi	1.9 ± 0.2	30.5 ± 2.5	*	0.05
ACMa-d	0.6 ± 0.1	67 ± 4.0	*	0.08
AOF-i	1 ± 0.15	13 ± 1.0	*	0.07
AFP-I	2.1 ± 0.2	16 ± 1.3	*	0.04
TFB	2.34 ± 0.3	8 ± 0.8	3 ± 0.5	0.02
ACABihem	4.3 ± 0.4	27 ± 2.2	*	0.01
AMCC	2.1 ± 0.25	40 ± 3.0	*	0.03
ACaM-D	4.3 ± 0.35	35 ± 2.5	*	0.05
ACaM-I	3.5 ± 0.3	34 ± 2.8	*	0.06
APec-d	2.8 ± 0.3	47 ± 3.5	*	0.07
APec-i	2.2 ± 0.2	43 ± 3.2	*	0.08

The A1-d and A1-i segments, which play a key role as primary inflow pathways for the anterior circulation, measured 4 ± 0.5 mm and 2 ± 0.3 mm in diameter, respectively. This difference was statistically significant (p = 0.01). The asymmetry in these segments could contribute to hemodynamic stress, increasing the likelihood of aneurysm formation, especially when a strong ACoA is absent. Previous studies on cerebral vascular anomalies have also linked similar disparities in diameter and length to aneurysm development.

Notably, the recurrent artery of Heubner (AR-Hubi) had an average diameter of 1.9 ± 0.2 mm and a length of 30.5 ± 2.5 mm, which aligns with findings from prior cadaveric studies. This artery plays a crucial role in anterior perforator syndromes, where its blockage can lead to ischemia in the caudate nucleus and the anterior limb of the internal capsule. Given its variability in shape and size, meticulous microsurgical dissection is essential, particularly in procedures such as ACoA aneurysm clipping and anterior skull base surgeries.

The bihemispheric anterior cerebral artery (ACABihem), measuring 4.3 ± 0.4 mm in diameter and 27 ± 2.2 mm in length, is a rare anatomical variant where one ACA supplies both hemispheres. This configuration is associated with an increased risk of ischemic strokes due to the reduced redundancy in collateral circulation. Identifying this variant preoperatively through MRA, CTA, or DSA can significantly improve surgical planning, particularly for bypass procedures and vascular reconstructions.

Additionally, the study highlights the surgical significance of the pericallosal and callosomarginal arteries (ACaM-D and ACaM-I). Their respective diameters (4.3 ± 0.35 mm and 3.5 ± 0.3 mm) and lengths (35 ± 2.5 mm and 34 ± 2.8 mm) are essential in understanding distal ACA aneurysms and stroke risks related to embolism or vasospasm. Preserving these arteries during anterior interhemispheric approaches is critical for preventing postoperative neurological deficits.

## Discussion

The findings of this study provide a detailed morphometric analysis of the azygos ACA, characterized by the absence of the ACoA and the presence of a singular A2 trunk. These observations enhance anatomical knowledge of this rare vascular variant and underline its clinical significance, particularly regarding aneurysm formation and surgical challenges. Our analysis offers updated insights into the unique vascular configuration and clinical implications of the azygos ACA.

Our results revealed the A1-d and A1-i segments, primary inflow pathways for anterior circulation, measuring 4 ± 0.5 mm and 2 ± 0.3 mm in diameter, respectively, with a statistically significant difference (p = 0.01). This asymmetry may contribute to hemodynamic stress and aneurysm formation, especially without an ACoA.

The recurrent artery of Heubner (AR-Hubi) measured 1.9 ± 0.2 mm in diameter and 30.5 ± 2.5 mm in length, consistent with prior cadaveric studies. Its occlusion can cause ischemia in the caudate nucleus and anterior internal capsule, emphasizing the need for careful microsurgical dissection, particularly in ACoA aneurysm clipping and skull base procedures.

ACABihem, measuring 4.3 ± 0.4 mm in diameter and 27 ± 2.2 mm in length, is a rare variant where one ACA supplies both hemispheres. This increases ischemic stroke risk due to reduced collateral circulation. Preoperative identification via MRA, CTA, or DSA is crucial for optimizing surgical planning, especially in bypass or vascular reconstruction.

The pericallosal and callosomarginal arteries (ACaM-D and ACaM-I), with diameters of 4.3 ± 0.35 mm and 3.5 ± 0.3 mm and lengths of 35 ± 2.5 mm and 34 ± 2.8 mm, are key in understanding distal ACA aneurysms and stroke risks. Their preservation in anterior interhemispheric approaches is vital to prevent neurological deficits.

Literature reports indicate an incidence of azygos ACA ranging from 0.3% to 1.6%, with de Sousa et al. [10] categorizing it as type IV in their classification of ACA anomalies. Similarly, Andreev et al. [11] noted the absence of the ACoA as a defining feature. While these studies provided foundational information, our detailed measurements of the A1 segments and branching patterns surpass prior data in accuracy and comprehensiveness [12].

Madkour [13] reported A2 trunk diameters similar to our findings (3.5 mm to 4.2 mm) but did not assess tapering or bifurcation points. Additionally, Ghanta et al. [14] associated azygos ACA with congenital anomalies such as CC agenesis; however, our specimen exhibited this variant without malformations. This aligns with the variability in clinical presentations described by Menshawi et al. [7], who explored diverse embryological origins and developmental pathways.

Our analysis of the branching patterns, including the pericallosal and callosomarginal arteries, builds on the work of Kashtiara et al. [15], who highlighted surgical implications but lacked precise morphometric data. Aneurysms related to the azygos ACA are of particular clinical concern, with a prevalence of up to 61% at junctions, as reported by Huh et al. [16]. Although rare, these aneurysms are clinically significant, warranting careful assessment during surgical planning.

A prenatal study of fetal brains older than four months identified the azygos ACA in only one of 60 cases, reflecting an incidence of 1.6% [9]. These findings emphasize the importance of considering this vascular anomaly during prenatal and postnatal evaluations due to its potential association with aneurysms [7,8,17].

Our methodological advancements, utilizing high-precision digital calipers (Mitutoyo Model CD-8” CX) and advanced photographic techniques, ensure unparalleled accuracy. This approach aligns with Rhoton’s [18] recommendations, emphasizing detailed anatomical studies to advance neurosurgical techniques.

The evolutionary and anatomical variability of the ACA is significant across species, increasing in complexity with evolutionary progression. In reptiles such as snakes, turtles, and crocodiles, the ACA is a single vessel, termed the "arteria ethmoidalis communis" by Rathke, from which vessels of the olfactory bulb originate [8,9,19]. In mammals, the ACA generally consists of two short vessels that converge into a median azygos artery, bifurcating into smaller branches to symmetrically supply the CC [9,20].

Embryologically, the ACA arises as the first branch of the internal carotid artery, forming multiple branches in embryos between 7-12 mm in size. The middle cerebral artery (MCA) emerges as a prominent branch in later stages (16-18 mm), with perforating branches influencing the development of anterior circulation [21,7]. The persistence of the azygos artery, often linked with anomalies such as agenesis of the CC, hydranencephaly, or AVMs, highlights its clinical complexity [22,23].

Diagnosis of azygos anterior cerebral artery

MRA provides visualization of cerebral arteries without contrast agents, ideal for detecting anomalies such as azygos ACA [19,23]. CTA combines advanced CT imaging with contrast dye to produce highly detailed visuals of vascular anomalies and related pathologies, aiding in accurate diagnosis [23-25]. DSA is recognized as the gold standard for vascular imaging, DSA provides precise localization and characterization of aneurysms using contrast dye and X-ray technology, making it invaluable for detailed assessments [23]. 3D rotational angiography generates three-dimensional reconstructions of the vascular system, offering critical insights for surgical planning and the evaluation of intricate vascular structures [3,26].

Early and accurate diagnosis is critical for effective management, enabling appropriate surgical or endovascular interventions to minimize the risk of complications associated with aneurysms and vascular anomalies [27-28].

Importance of cadaveric studies

Cadaveric studies remain crucial for understanding anatomical variations and their clinical implications. They allow for detailed dissection and morphometric analysis, providing data that is often challenging to obtain in vivo [29-31]. These studies document rare variants, such as the azygos ACA, and enhance surgical and radiological practice by offering insights into vascular anomalies. Moreover, cadaveric studies provide an invaluable educational tool, fostering a hands-on understanding of anatomical complexity [32-33].

Limitations of this study

While this study offers valuable insight into the morphometric features of the azygos anterior cerebral artery, several limitations should be considered when interpreting our findings. First and foremost, this analysis is based on a single anatomical specimen, which naturally limits the ability to generalize the observations. Although the dissection was performed with high precision, we recognize that broader anatomical patterns require analysis across multiple specimens or patient populations. Secondly, we did not include a control group for comparison. Without parallel measurements from a brain with a conventional ACA configuration, it becomes more difficult to place our findings within the full spectrum of anatomical variation. Future work comparing different configurations would strengthen the relevance of this type of study.

Another important point is the nature of post-mortem changes. The use of formalin fixation and latex injection, while necessary for preservation and visualization, can slightly alter the natural elasticity and dimensions of cerebral vessels. We attempted to minimize these effects with careful technique, but minor distortions are still possible. Although measurements were made using high-resolution digital tools, human error in the dissection and measurement process cannot be entirely ruled out. We took several steps to verify our results, including repeated measurements and documentation with graph paper and high-resolution photography, but manual handling always introduces a degree of variability.

In addition, our focus was limited to the anterior cerebral circulation, which means we may have missed associated vascular anomalies in the middle or posterior territories that could be relevant in a clinical setting, especially in patients with complex cerebrovascular presentations. Finally, while the anatomical insights are significant, this study lacks a functional or clinical correlation. The cadaveric nature of the research means we could not assess how this variant might influence cerebral blood flow or relate directly to patient outcomes. Integrating imaging data or clinical case series would help bridge this gap in future studies.

## Conclusions

This cadaveric study emphasizes the azygos ACA as an uncommon but clinically significant vascular variation. Key findings include the absence of the ACoA and the presence of a single A2 trunk, which may increase susceptibility to aneurysm development and complicate surgical procedures. Understanding these rare variations is essential for accurate diagnosis, effective surgical planning, and minimizing associated risks. While this analysis is based on a single case, it highlights the value of advanced imaging techniques and cadaveric studies in deepening our knowledge of neurovascular anomalies and enhancing patient care.
